# Psychometric properties of the Questionnaire for the Use of Public Transportation and Well-Being: Bayesian reliability, factorial invariance, and structural equation modeling evidence

**DOI:** 10.3389/fsoc.2026.1791429

**Published:** 2026-05-05

**Authors:** Jonatan Baños-Chaparro, Tomás Caycho-Rodríguez, Diego Valencia-Pecho, Esteban Sarmiento-Suarez, Dulce Bernabel-Tarazona, Gabriela Rivera-Álvarez

**Affiliations:** 1 Programa académico de Psicología, Facultad de Ciencias de la Salud, Universidad Privada Norbert Wiener, Lima, Peru; 2 Facultad de Psicología, Universidad Científica del Sur, Lima, Peru

**Keywords:** adults, mental health, psychometric validation, public transport, urban mobility

## Abstract

**Introduction:**

Public transport is a fundamental component of urban mobility and an everyday context that can influence the emotional well-being of adults. However, there is limited availability of validated psychometric instruments that specifically assess well-being associated with public transport use in adults. Within this framework, the Questionnaire for the Use of Public Transportation and Well-Being (QUPTW) constitutes a relevant tool whose psychometric performance needs to be examined across different cultural contexts, particularly within specific local settings such as Latin American urban populations.

**Objective:**

To analyse the psychometric properties of the QUPTW in adults in Metropolitan Lima, Peru.

**Methods:**

The study design was psychometric, with a quantitative, basic, and cross-sectional approach. A total of 507 adults from Metropolitan Lima, Peru (66.7% women) participated. A sociodemographic questionnaire and the psychological instruments QUPTW, Dimensions of Anger Reactions-5 (DAR-5), Patient Health Questionnaire-2 (PHQ-2), and Generalized Anxiety Disorder-2 (GAD-2) were administered. Structural equation modeling and Bayesian reliability were used for statistical analysis.

**Results:**

The findings indicated that the QUPTW demonstrates adequate content validity (*V* > 0.70), a unidimensional structure [CFI = 0.99, RMSEA = 0.07 (90% CI: 0.055, 0.086), SRMR = 0.04], and good reliability (*ω* = 0.88 and *H* = 0.93). In addition, invariance by sex was observed, as well as associations with generalised anxiety (*r* = 0.27, *p* = 0.001), depressive symptoms (*r* = 0.21, *p* = 0.001), and anger (*r* = 0.47, *p* = 0.001).

**Conclusion:**

The results support the QUPTW as a valid, reliable, and sex-invariant instrument for assessing emotional well-being associated with public transport use in adults in Metropolitan Lima, Peru. These findings provide evidence of its adequacy within this specific urban Latin American context and should be interpreted with caution when generalising to other cultural settings. Its application is useful both for research purposes and for the design of interventions aimed at promoting mental health and improving quality of life in similar urban contexts.

## Introduction

Public transport plays a central role in contemporary societies, particularly in urban contexts, where it constitutes one of the main means of everyday mobility for the adult population. Globally, a considerable proportion of adults relies on buses, trains, metro systems and other forms of collective transport to travel to work, education and social activities (Iamtrakul et al., 2024; Tuvikene et al., 2023). The growing urbanisation of cities, traffic congestion, environmental concerns and economic constraints have further reinforced the relevance of public transport as a sustainable alternative to private vehicle use (de Oña, 2022). From an urban sociology perspective, public transport not only fulfils an instrumental function but also constitutes an everyday urban space in which social inequalities, implicit norms and shared emotional experiences are articulated (Fayard, 2025; Tuvikene et al., 2023). In many low- and middle-income countries, including those in Latin America, public transport is not only widely used but is often the primary or sole option for adult mobility, making it a key contextual factor shaping everyday experiences and quality of life (Kaiser and Barstow, 2022; Tiznado-Aitken et al., 2023).

Beyond its instrumental function, the use of public transport has attracted growing interest in relation to mental health. Daily commuting in crowded, noisy and unpredictable transport environments has been associated with various mental health difficulties, including increased stress, anxiety, depressive symptoms and anger (Conceição et al., 2023; Iamtrakul et al., 2024; Lan et al., 2020; Ye et al., 2023). In adults, generalised anxiety may arise from uncertainty, perceived lack of control, safety concerns and time pressure during journeys (Lan et al., 2020). Depressive symptoms may be linked to chronic exposure to aversive commuting conditions, fatigue and a reduced sense of autonomy, while anger may emerge from overcrowding, delays, interpersonal conflicts and violations of personal space (Conceição et al., 2023; Ye et al., 2023). These experiences can be understood through perspectives from urban environmental psychology and daily stress models, which highlight how urban environmental characteristics cumulatively influence emotional well-being (Conceição et al., 2023; Kou et al., 2021). Such emotional responses may be exacerbated by long travel times and poor service quality, suggesting that public transport is not merely a logistical issue but also a psychologically meaningful context that can influence emotional well-being (Iamtrakul et al., 2024).

Recent studies have further expanded this perspective by linking travel behavior with broader health outcomes. For instance, participation in activity-related travel and higher levels of physical activity during commuting have been associated with improvements in overall health and well-being (Ali et al., 2024a). In addition, research on travel mode choice has demonstrated that selecting more sustainable modes of transport is associated with better health parameters and improved well-being outcomes (Ali et al., 2024a). Similarly, studies examining activity-travel participation and multitasking during travel highlight the complex ways in which daily mobility patterns influence subjective well-being (Ali et al., 2023). Together, these findings emphasise that public transport experiences are embedded within broader behavioral and health-related processes, reinforcing the need to assess not only functional aspects of mobility but also their emotional and psychological implications.

It is important to note that experiences of public transport use and their mental health correlates are not uniform across individuals, with relevant differences observed between women and men. Women tend to report higher levels of anxiety and a greater perception of insecurity when using public transport, which may be related to greater exposure to harassment, fear of victimisation and gendered social norms associated with safety and vulnerability in public spaces (Alfaro et al., 2024; Kacharo et al., 2022). Men, in contrast, may exhibit higher levels of anger or irritability, possibly linked to social expectations regarding emotional expression, competitiveness and tolerance of frustration (Conceição et al., 2023; Mackett, 2022). These differences reflect patterns of differentiated use of urban space and unequal exposure to risks and stressors during everyday mobility, underscoring the importance of examining factorial invariance and ensuring that psychological measures related to public transport function equivalently for women and men, thereby allowing valid comparisons and appropriate interpretations of mental health outcomes.

Within this framework, the assessment of emotional well-being associated with public transport use is particularly relevant. Self-report psychological instruments constitute an efficient and theoretically grounded strategy for capturing subjective experiences, emotional reactions and perceived impacts of public transport on well-being. The literature has developed various scales aimed at assessing specific aspects of commuting, such as the Satisfaction with Travel Scale (STS; Ettema et al., 2011), which focuses on cognitive and affective satisfaction with travel, or the Multimodal Commuting Stress Scale (MCSS; Useche et al., 2023), designed to measure stress associated with different modes of transport. Likewise, Fear of Crime in Public Transport Scales have made it possible to assess perceived insecurity and fear of crime in public transport contexts (Vilalta, 2011). Taken together, these instruments have made significant contributions to the study of urban mobility; however, their scope is limited to partial dimensions of the travel experience and they do not provide a comprehensive assessment of emotional well-being associated with public transport use (Ettema et al., 2011; Useche et al., 2023; Vilalta, 2011). Moreover, recent evidence linking mobility patterns with health and well-being highlights the need for integrative measures capable of assessing emotional responses in real-life commuting contexts (Ali et al., 2023, 2024a,b). This reveals a clear research gap in the availability of comprehensive and psychometrically robust instruments specifically designed to assess emotional well-being associated with public transport use. In this context, the Public Transport Use and Well-being Questionnaire (QUPTW) was developed to address this gap, offering a multidimensional assessment of emotional well-being specifically linked to public transport use (Torales et al., 2024). Having a valid measure of these experiences is crucial for informing urban policies aimed at sustainable mobility and public health. The adaptation and validation of the QUPTW in the Peruvian context are particularly relevant, given the distinctive characteristics of public transport systems, sociocultural norms and urban stressors present in Peru, which may differentially shape users’ emotional responses (Stiglich, 2021; Valderrama Reyes and Florián Plasencia, 2022; Zarzosa Marquez et al., 2024).

Accordingly, the present study aims to examine the psychometric properties of the QUPTW in a sample adults in Metropolitan Lima, Peru. Specifically, the study pursues the following objectives: (1) to analyse the content validity of the QUPTW; (2) to assess the reliability of the instrument; (3) to examine its internal structure; (4) to test its factorial invariance by sex; and (5) to analyse its relationship with other theoretically relevant variables. In line with these objectives, it is expected that the QUPTW will demonstrate adequate content validity, adequate reliability, a coherent internal structure, evidence of factorial invariance across sex, and positive relationships with anger, depressive symptoms, and anxiety. In doing so, the study aims to offer a robust and culturally relevant measure, contributing to the advancement of research in urban sociology and supporting the design of interventions aimed at improving mental health and quality of life among adult users of public transport.

## Materials and methods

### Design

The study corresponds to an instrumental design, in which the psychometric properties of the QUPTW are analysed (Ato et al., 2013). Additionally, it is a basic, cross-sectional study with a quantitative approach, aimed at examining the validity and reliability of the instrument in a sample of adults from Metropolitan Lima, Peru.

### Participants

The study sample consisted of adults from Metropolitan Lima, Peru, an exclusively urban setting. The sample was selected using non-probability convenience sampling. The inclusion criteria were as follows: (a) being 18 years of age or older, (b) residing in Metropolitan Lima (urban area), and (c) providing informed consent. The exclusion criteria were as follows: (a) having a diagnosis of an intellectual or neurological developmental disorder, (b) questionnaires with missing responses in the sociodemographic section and psychological instruments, and (c) voluntary withdrawal from the survey. Given the geographic scope of the study, only urban participants were included; therefore, rural and peri-urban populations were not part of the sampling frame. Accordingly, a total of 507 adults participated, with women representing the majority of the participants (66.7%). The mean age of the sample was 33 years (SD = 12.05), with ages ranging from 18 to 60 years. In relation to employment status, most respondents indicated that they were currently employed (72.4%), whereas 27.6% reported being unemployed at the time of the survey. Regarding marital status, the largest proportion of participants reported being single (72%), followed by those who were married (25%). Smaller percentages identified as divorced (2.8%) or widowed (0.2%). As expected, all participants resided in urban areas (95.9%) due to the study being conducted exclusively in Metropolitan Lima. With respect to educational attainment, incomplete university education was the most frequently reported level (32.7%), followed by completed university studies (26.2%) and completed technical education (12.2%), reflecting a predominantly educated urban adult sample.

### Measures

#### Sociodemographic questionnaire

A brief demographic questionnaire was used to gather information on gender, age, employment status, marital status, area of residence, and educational level.

#### Questionnaire on the Use of Public Transportation and Well-Being (QUPTW)

This is a nine-item questionnaire that assesses perceptions of public transportation use and its relationship with well-being (Torales et al., 2024). Each item is rated using a five-point Likert scale ranging from 0 (strongly disagree) to 4 (strongly agree). The total score ranges from 9 to 45, with higher scores indicating poorer well-being. Given that the scale is available in Spanish (Torales et al., 2024), the present study conducted content validity through expert judges and a pilot sample, as well as an examination of its psychometric properties, which are reported in the Results section.

#### Dimensions of Anger Reactions-5 (DAR-5)

The DAR-5 is a five-item scale that evaluates experiences of anger over the past 4 weeks. It uses a five-point Likert scale ranging from 1 (none or almost none of the time) to 5 (all or almost all of the time). Total scores range from 5 to 25, with higher scores indicating more frequent experiences of anger. The Peruvian adaptation was used, and this study showed adequate reliability (*ω* = 0.86) (Caycho-Rodríguez et al., 2020).

#### Patient Health Questionnaire-2 (PHQ-2)

The PHQ-2 is a brief two-item questionnaire that assesses depressive symptoms over the past 2 weeks. Each item is rated on a four-point scale from 0 (not at all) to 3 (nearly every day). The total score ranges from 0 to 6, with higher scores indicating greater depressive symptomatology. The Peruvian adaptation was used, and this study reported good reliability (*ω* = 0.73) (Baños-Chaparro et al., 2021).

#### Generalized Anxiety Disorder-2 (GAD-2)

The GAD-2 is a short scale assessing generalized anxiety over the past 2 weeks through two items. Each item is rated on a four-point scale from 0 (not at all) to 3 (nearly every day). The total score ranges from 0 to 6, with higher scores reflecting greater levels of generalized anxiety. The Peruvian adaptation was used, and this study reported adequate reliability (*ω* = 0.88) (Baños-Chaparro, 2022).

### Procedure

Data were collected through an online self-administered survey conducted between January and April 2025 using the Google Forms platform. The questionnaire was disseminated via the researchers’ social media networks, enabling broad outreach and facilitating participation from individuals in diverse geographic and social contexts.

Prior to participation, respondents were provided with detailed information regarding the study’s objectives, its exclusively academic purpose, the voluntary nature of participation, procedures for data management, and assurances of anonymity and confidentiality. Informed consent was obtained electronically from all participants before accessing the survey items, in accordance with ethical standards for research involving human subjects.

The use of internet-based survey methods was strategically selected due to their demonstrated advantages in social science research, including enhanced accessibility to heterogeneous samples, systematic control of data collection procedures, flexibility in incorporating diverse response formats, and increased efficiency in terms of time and cost. Furthermore, online surveys reduce logistical constraints associated with traditional data collection methods while maintaining acceptable levels of data quality and reliability, as documented in previous methodological literature (Gosling and Mason, 2015).

### Statistical analysis

The statistical analysis was conducted in a sequential and systematic manner using RStudio software (version 4.3.2), following a multi-stage approach aimed at the comprehensive psychometric evaluation of the instrument.

In the first stage, a thorough descriptive analysis of the items was performed to examine their statistical behavior and preliminary quality. Measures of central tendency and dispersion (mean and standard deviation) were calculated, along with distributional indicators such as skewness and kurtosis. In addition, a polychoric correlation matrix was estimated, taking into account the ordinal nature of the items, together with corrected item–total correlations, adopting values greater than 0.30 as the criterion for adequacy. Complementarily, content validity was assessed using Aiken’s *V* coefficient, with values above 0.70 considered acceptable, in accordance with previous methodological recommendations (Kline, 2023; Roebianto et al., 2023).

In the second stage, a confirmatory factor analysis (CFA) was conducted to examine the internal structure of the instrument. Given the ordinal nature of the data, the Diagonally Weighted Least Squares (DWLS) estimator was employed, as it is considered appropriate for this type of measurement. Model fit was evaluated using multiple goodness-of-fit indices, including the Comparative Fit Index (CFI), the Root Mean Square Error of Approximation (RMSEA), and the Standardised Root Mean Square Residual (SRMR). Values of CFI greater than 0.95 and RMSEA and SRMR values below 0.08 were used as criteria for acceptable model fit. Additionally, the magnitude of the standardised factor loadings was examined, with values exceeding 0.30 considered adequate (Jordan Muiños, 2021).

The third stage focused on estimating the reliability of the instrument from a contemporary perspective. The Bayesian omega coefficient (*ω*) and the *H* coefficient were computed. This approach allowed for more precise and robust estimates of score consistency, overcoming the limitations of traditional reliability coefficients, as recommended in the specialised literature (Baños-Chaparro and Caycho-Rodríguez, 2024; Gunnell, 2020).

In the fourth stage, factorial invariance by sex was examined, again using the DWLS estimator. Initially, a configural model was tested to assess the equivalence of the factorial structure of the QUPTW between women and men. Subsequently, constraints were introduced progressively to test successive levels of invariance: thresholds (threshold invariance), factor loadings (metric invariance), intercepts (scalar invariance), and residuals (strict invariance). Model comparisons were conducted using criteria based on minimal changes in fit indices, considering ΔCFI < 0.010 and ΔSRMR ≤ 0.030 as evidence of acceptable invariance (Chen, 2007; Finch and French, 2018).

Finally, in the fifth stage, a covariance-based structural equation model (CB-SEM) was estimated to examine the relationships among the latent variables in the study. The robust maximum likelihood (MLR) estimator was employed, and overall model fit was assessed using the CFI, RMSEA, and SRMR indices, following the same acceptance criteria previously established (Jordan Muiños, 2021). The magnitude of the associations was interpreted according to the cut-off values proposed by Cohen (1988), considering small = 0.10, moderate = 0.30, and large = 0.50 effects.

### Ethical considerations

The research was conducted in strict compliance with international and national ethical standards governing psychological and social science research. Prior to data collection, all participants were informed about the nature and purpose of the study and provided their informed consent electronically. Participation was entirely voluntary, the survey was administered in an anonymous format, and robust measures were implemented to ensure the confidentiality and secure handling of all collected data, in line with established ethical guidelines (Hilbig et al., 2022). Furthermore, the study protocol underwent formal ethical review and received approval from the Ethics Committee of Universidad Privada Norbert Wiener, under registration number 0833-2024-CIEIC-UPNW, thereby confirming that the research procedures met the required ethical and institutional standards for studies involving human participants.

## Results

### Evidence based on content

The expert judges evaluated the content of the items according to the criteria of relevance, representativeness, and clarity, with Aiken’s *V* values exceeding 0.70 (Table 1). All expert judges (*n* = 5) approved the final version of the QUPTW. Likewise, in the pilot sample (*n* = 7), no suggestions or modifications were made.

**Table 1:** Content validity of the QUPTW items.

Items	Relevance (*n* = 5)		Representativeness (*n* = 5)		Clarity (*n* = 5)	
	*V*	CI 95%	*V*	CI 95%	*V*	CI 95%
1	0.83	0.65, 0.93	0.83	0.65, 0.93	0.83	0.65, 0.93
2	1.00	0.87, 1.00	1.00	0.87, 1.00	1.00	0.87, 1.00
3	0.93	0.75, 0.99	0.73	0.52, 0.87	0.73	0.52, 0.87
4	0.87	0.67, 0.95	0.93	0.75, 0.99	0.87	0.67, 0.95
5	0.73	0.52, 0.87	0.87	0.67, 0.95	0.93	0.75, 0.99
6	0.93	0.75, 0.99	0.73	0.52, 0.87	0.73	0.52, 0.87
7	0.73	0.52, 0.87	0.93	0.75, 0.99	0.80	0.59, 0.92
8	0.87	0.67, 0.95	0.93	0.75, 0.99	0.87	0.67, 0.95
9	0.80	0.59, 0.92	0.87	0.67, 0.95	0.93	0.75, 0.99

*V*, Aiken’s *V*, CI, confidence intervals.

### Descriptive analysis

Table 2 shows that the highest arithmetic mean was observed for item 6 (*M* = 3.97), while the lowest was found for item 7 (*M* = 3.17). With regard to variability, the greatest standard deviation occurred in item 9 (SD = 1.24) and the smallest in item 2 (SD = 0.97). The skewness and kurtosis values fell within the ±1.5 range. In addition, all corrected item–total correlations were satisfactory, exceeding 0.30. Finally, the polychoric correlation matrix indicated positive relationships, with no evidence of multicollinearity (*r* > 0.90).

**Table 2:** Descriptive measures and correlation matrix.

Item	*M*	SD	*g*_1_	*g*_2_	*r*_it_	Polychoric correlation matrix								
1	3.64	1.04	−0.67	0.04	0.61	–								
2	3.75	0.97	−0.78	0.36	0.59	0.58	–							
3	3.34	1.05	−0.46	−0.26	0.74	0.64	0.60	–						
4	3.66	0.99	−0.79	0.19	0.73	0.59	0.55	0.68	–					
5	3.39	1.01	−0.46	−0.24	0.73	0.58	0.48	0.75	0.72	–				
6	3.97	1.03	−1.01	0.55	0.62	0.40	0.52	0.55	0.63	0.54	–			
7	3.17	1.06	−0.05	−0.64	0.68	0.46	0.43	0.65	0.61	0.68	0.60	–		
8	3.62	0.98	−0.72	0.25	0.74	0.56	0.55	0.63	0.66	0.68	0.58	0.72	–	
9	3.42	1.24	−0.39	−0.81	0.31	0.21	0.17	0.21	0.24	0.24	0.20	0.25	0.38	–

*M* = media, SD = standard deviation, *g*_1_ = skewness, *g*_2_ = kurtosis, *r*_it_ = corrected item test correlation.

### Evidence based on internal structure

**Figure 1:**
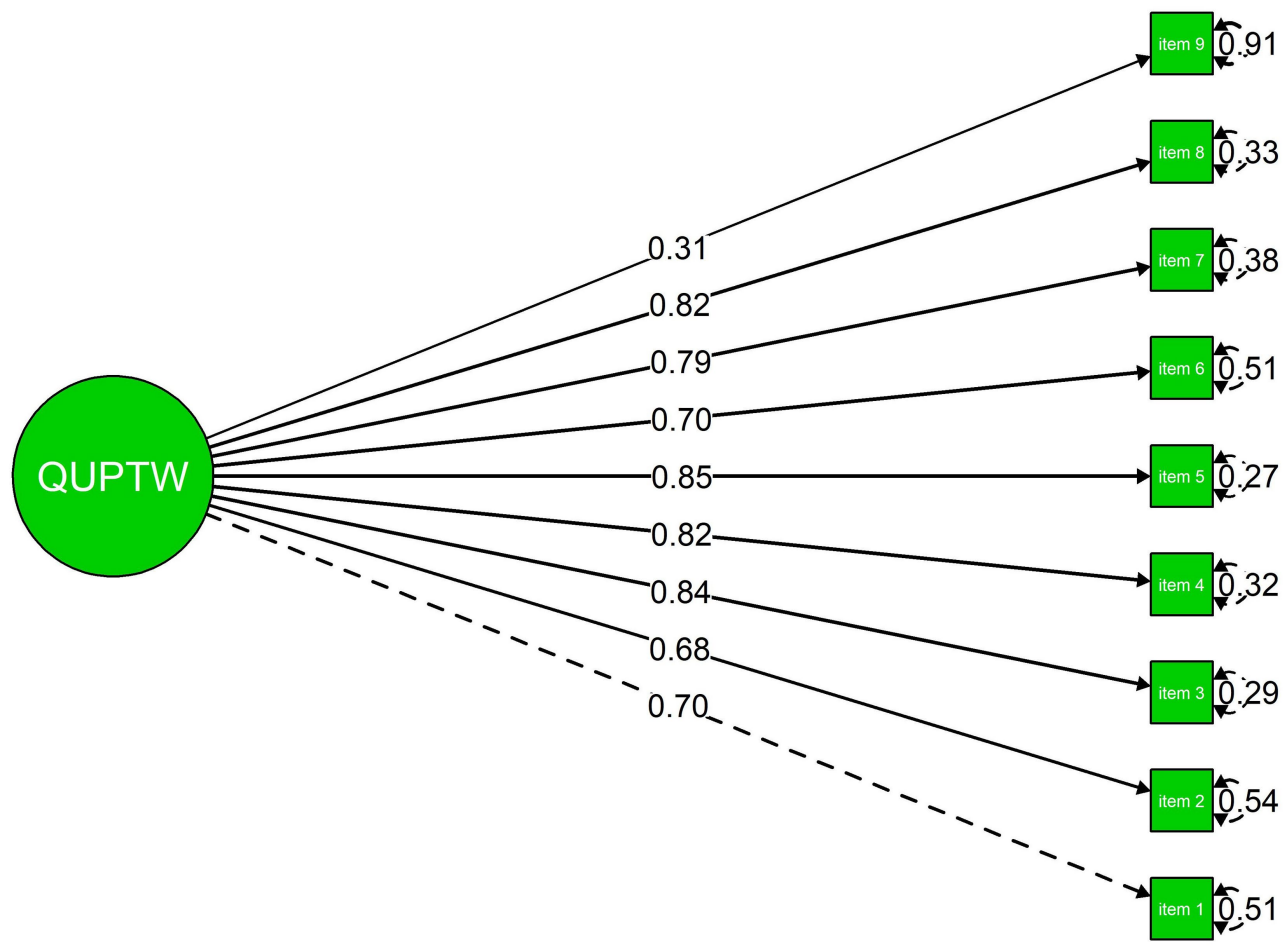
Factor structure of the QUPTW.

The confirmatory factor analysis supported a unidimensional structure of the QUPTW, showing good model fit [CFI = 0.99, RMSEA = 0.07 (90% CI: 0.055, 0.086), SRMR = 0.04]. Standardised factor loadings ranged from 0.31 to 0.85, indicating that all items contributed meaningfully to the latent construct (Figure 1).

### Reliability

The posterior estimate of the *ω* coefficient was 0.88, with a 95% credibility interval ranging from 0.86 to 0.89, indicating a 95% probability that the true value of ω lies within this range. Likewise, the reliability coefficients *H* = 0.93 considered adequate.

#### Measurement invariance

Table 3 presents the results of the factorial invariance analysis. The configural model does not differ significantly from the more restrictive models, that is, those imposing constraints on factor loadings, thresholds, intercepts, and residuals, as the changes in fit indices (ΔCFI < 0.010, ΔSRMR < 0.030) remain within acceptable limits. These results support the invariance of the QUPTW across women and men.

**Table 3:** Invariance analysis of the QUPTW by sex.

Models	*X*^2^ (gl)	*p*	CFI	RSMR	∆CFI	∆RSMR
M1	110.43(54)	0.001	0.924	0.048	–	–
M2	116.26(72)	0.001	0.924	0.048	0.000	0.000
M3	130.90(80)	0.001	0.929	0.049	0.005	0.001
M4	176.56(88)	0.001	0.932	0.049	0.003	0.000
M5	189.07(97)	0.001	0.941	0.053	0.009	0.004

M1 = configural invariance, M2 = threshold invariance, M3 = metric invariance, M4 = scalar invariance, M5 = strict invariance.

### Evidence based on the relationship with other variables

**Figure 2:**
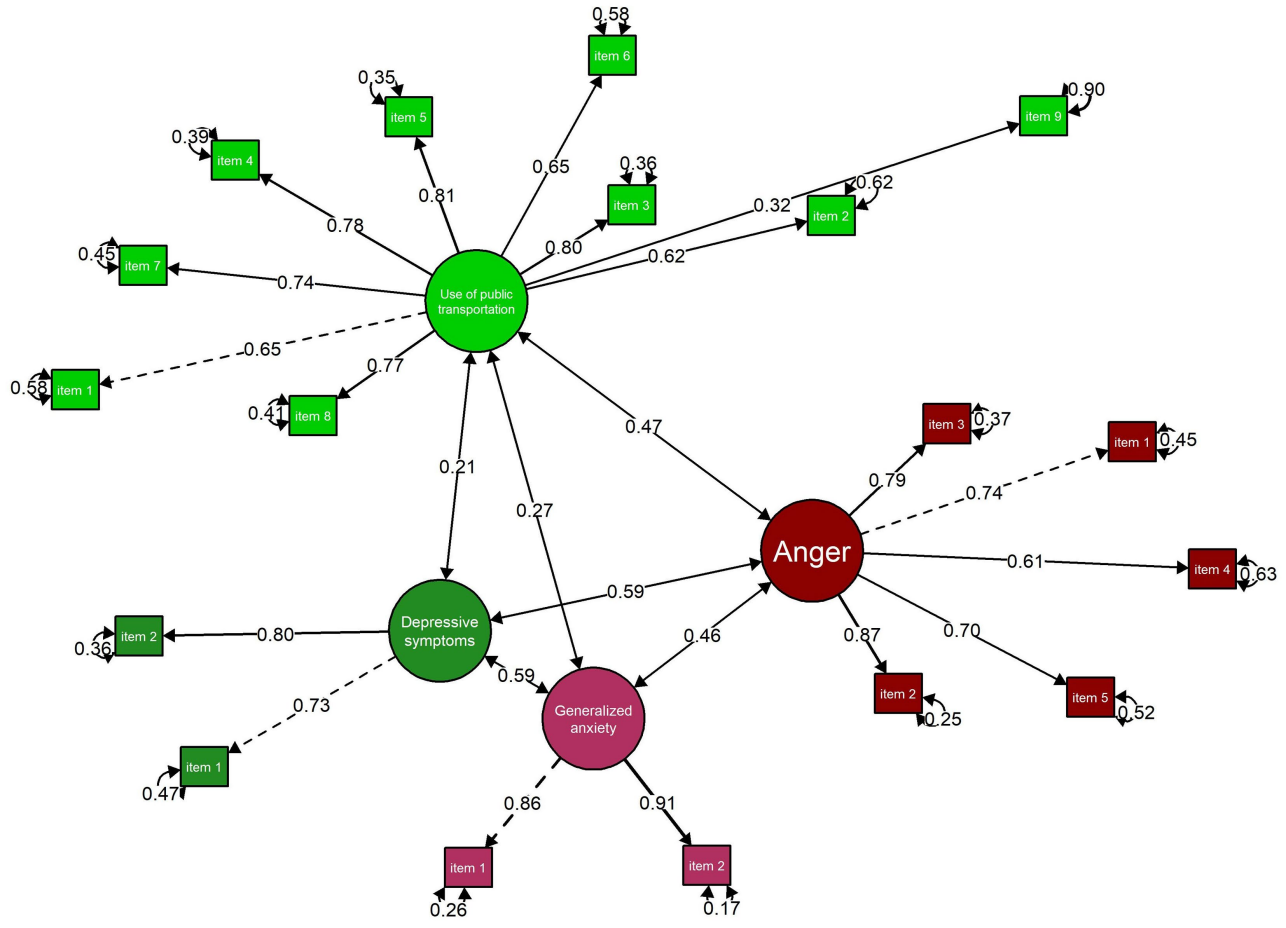
Structural model of the relationship between use of public transportation, anger, depressive symptoms, and generalized anxiety.

Figure 2 illustrates the results of the correlation analysis examining the associations between use of public transportation and the psychological variables included in the model. The estimated structural equation model showed an adequate fit to the data, as indicated by the goodness-of-fit indices (CFI = 0.94, RMSEA = 0.05, 90% CI [0.050, 0.063], and SRMR = 0.04), supporting the robustness of the proposed relational structure.

The findings revealed that use of public transportation was positively and statistically significantly associated with all the psychological outcomes examined. In particular, a moderate association was observed with anger (*r* = 0.47, *p* = 0.001), whereas small associations were identified with depressive symptoms (*r* = 0.21, *p* = 0.001) and generalised anxiety (*r* = 0.27, *p* = 0.001).

## Discussion

The present study aimed to examine the psychometric properties of the QUPTW in a sample of adults in Metropolitan Lima, Peru, addressing the need for valid and reliable instruments to assess emotional well-being associated with the everyday use of public transport. In contemporary urban contexts, public transport not only constitutes a means of mobility but also an everyday social space where environmental stressors, social interactions and emotional experiences converge, potentially exerting a significant influence on mental health (Fayard, 2025; Tuvikene et al., 2023). In this regard, the availability of robust psychometric measures is essential for advancing understanding of the impact of public transport on adults’ emotional well-being, as well as for informing public policies and urban health intervention strategies. The results provide empirical evidence regarding the psychometric performance of the QUPTW in this context and support its use for assessing emotional well-being associated with urban mobility among adults in Metropolitan Lima, Peru.

These findings are consistent with recent research highlighting the role of daily mobility patterns in shaping physical and psychological health outcomes. For instance, studies have shown that travel behavior, including activity-travel participation and mode choice, is closely linked to subjective well-being and health indicators (Ali et al., 2023, 2024a,b). In line with this evidence, the present study reinforces the idea that public transport is not merely a functional activity but a context with meaningful implications for emotional well-being. However, unlike these studies, which focus primarily on behavioral and health outcomes, the present study contributes by providing a psychometrically validated instrument specifically designed to assess the emotional dimension of public transport use.

With regard to factorial structure, the results showed that the QUPTW presents a unidimensional structure, indicating that the items of the instrument assess a global construct of emotional well-being linked to public transport use. This finding is consistent with the original study on the development of the QUPTW, which also reported a unidimensional factorial structure, suggesting conceptual stability of the construct across different cultural contexts (Torales et al., 2024). Replication of this structure in the Peruvian population reinforces the structural validity of the instrument and supports its usefulness for parsimonious and coherent assessment of emotional experiences associated with public transport. Furthermore, a unidimensional structure facilitates score interpretation and application in both research and applied settings, where clear and efficient measurement of emotional well-being is required (Kou et al., 2021).

From a comparative perspective, the unidimensional structure identified in this study aligns with previous research suggesting that emotional responses to mobility environments tend to be experienced as a global psychological state rather than as strictly independent dimensions (Iamtrakul et al., 2024). Nevertheless, some studies have conceptualised travel-related well-being as a multidimensional construct, incorporating cognitive, affective and behavioral components (Ettema et al., 2011). This difference may be explained by the specific focus of the QUPTW on emotional well-being in public transport contexts, which may favour a more integrated representation of emotional experiences.

Regarding reliability, the QUPTW showed adequate values using both the Bayesian omega coefficient and coefficient *H*, indicating high precision in measuring the latent construct. These results are consistent with the evidence reported in the original QUPTW study, which documented satisfactory reliability indicators using contemporary approaches (Torales et al., 2024). The use of Bayesian reliability estimates represents a methodological strength, as it allows for more robust estimates that are less dependent on strict assumptions, particularly in scales with ordinal items (Pfadt et al., 2022). Taken together, these findings suggest that the QUPTW provides consistent and reliable scores, reinforcing its suitability for assessing emotional well-being associated with public transport use among adults in Metropolitan Lima, Peru.

A distinctive contribution of the present study is the assessment of measurement invariance of the QUPTW by sex, an aspect not addressed in the original instrument development study (Torales et al., 2024). The results showed that the QUPTW demonstrates measurement invariance between women and men, indicating that both the items and the underlying construct are interpreted equivalently across groups. This finding is particularly relevant in the context of public transport, where everyday mobility experiences often differ between women and men due to factors such as perceived safety, exposure to stressors and gendered social norms (Alfaro et al., 2024; Conceição et al., 2023; Kacharo et al., 2022; Mackett, 2022). By demonstrating that the QUPTW functions equivalently across sex, the present study substantially expands the available psychometric evidence and supports the use of the instrument for making valid comparisons between women and men in adults in Metropolitan Lima, Peru. In doing so, it reduces the risk of interpreting observed differences as reflecting genuine inequalities when they may instead be attributable to measurement bias.

This result is also consistent with broader evidence indicating that, although men and women may differ in the intensity or type of emotional responses during commuting, the underlying psychological constructs can be measured equivalently when instruments are properly specified (Mackett, 2022). However, future research could further explore potential differences at the latent mean level to better understand gender-based disparities in emotional experiences related to public transport.

With respect to evidence based on relationships with other variables, the results showed significant associations between emotional well-being as assessed by the QUPTW and relevant mental health variables, such as generalised anxiety, depressive symptoms and anger. These relationships are consistent with the literature documenting the impact of daily commuting on adults’ emotional states. It is plausible that adverse travel conditions, such as overcrowding, service unpredictability and perceived insecurity, contribute to increased anxiety and irritability (Iamtrakul et al., 2024; Lan et al., 2020; Ye et al., 2023), while chronic exposure to negative experiences during daily mobility may be related to depressive symptoms (Conceição et al., 2023). These findings support the convergent validity of the QUPTW and confirm that the instrument captures an emotionally relevant construct linked to established mental health indicators in adults in Metropolitan Lima, Peru.

In addition, recent studies have shown that travel behavior and mode choice are associated with subjective well-being and health outcomes (Ali et al., 2023, 2024a). The present findings are consistent with this body of evidence, as they demonstrate that emotional responses to public transport are significantly linked to key mental health indicators. However, while previous studies have primarily focused on behavioral predictors of well-being, the present study advances the field by operationalising emotional well-being as a measurable construct within a psychometric framework.

The implications of the study can be considered from both theoretical and practical perspectives. From a theoretical standpoint, the QUPTW contributes to the fields of transport psychology and urban sociology by operationalising emotional well-being as a construct linked to everyday mobility experiences. The instrument enables the integration of perspectives from environmental psychology, urban stress and daily mobility, providing an empirical tool to examine how public transport conditions influence mental health. This perspective is consistent with prior research that conceptualises public transport as a social and experiential space shaping everyday urban life (Fayard, 2025; Tuvikene et al., 2023). These contributions are aligned with recent research emphasising the need for integrative approaches that connect mobility patterns, environmental exposures and psychological outcomes (Ali et al., 2024a; Iamtrakul et al., 2024). In this sense, the QUPTW offers a novel contribution by providing a standardised tool capable of capturing these complex interactions from an emotional perspective. From a practical perspective, the QUPTW offers a valuable resource for researchers, urban planners and policy-makers interested in assessing the emotional impact of public transport on adults in Metropolitan Lima, Peru. In the Peruvian context, characterised by structural challenges in public transport systems, including service quality issues and urban infrastructure constraints (Stiglich, 2021; Valderrama Reyes and Florián Plasencia, 2022), the use of the QUPTW may facilitate the identification of factors associated with users’ emotional well-being.

The present study has several methodological strengths, including the estimation of Bayesian reliability and the novel psychometric contributions of the QUPTW in terms of measurement invariance and relationships with other variables. Nevertheless, some limitations should also be considered. First, the use of non-probabilistic sampling restricts the generalisability of the findings; future research should employ probabilistic sampling designs or stratified sampling strategies to ensure greater representativeness of different sociodemographic groups. Second, the cross-sectional design precludes the establishment of causal relationships between public transport use and mental health variables; longitudinal studies are therefore needed to examine the temporal stability of the construct and its evolution over time. Additionally, the observed sampling bias, characterised by greater participation of women and urban residents, suggests the need for more balanced samples and the inclusion of rural or peri-urban populations in future studies. Furthermore, test–retest reliability was not assessed; future research should therefore examine the temporal stability of the QUPTW. Finally, the use of complementary approaches, such as item response theory, would allow for more precise analysis of item functioning, identification of potential differences in discrimination and difficulty, and further strengthening of the instrument’s psychometric evidence.

## Conclusion

The results of the present study support the validity and reliability of the QUPTW as a unidimensional, precise, and sex-invariant measure for assessing emotional well-being associated with public transport use among adults in Metropolitan Lima, Peru. Specifically, the findings confirm the internal structure of the instrument, its adequate reliability, measurement invariance across sex, and its theoretically coherent relationships with relevant mental health variables such as anxiety, depressive symptoms, and anger.

Overall, the present study provides robust psychometric evidence supporting the use of the QUPTW in adults in Metropolitan Lima, Peru, contributing to its contextual validation in this population. In particular, the QUPTW emerges as a relevant tool for the study of emotional well-being among adults in urban contexts such as Metropolitan Lima, contributing to the advancement of scientific knowledge and to the design of interventions aimed at improving quality of life and mental health among public transport users.

## Data Availability

The raw data supporting the conclusions of this article will be made available by the authors, without undue reservation.
